# Distance-based ratio metric assay using thread-based devices for Karaya gum detection in food samples

**DOI:** 10.1038/s41598-025-19210-4

**Published:** 2025-09-29

**Authors:** Reshmi Saravana Bhava, Anusha Prabhu, Balachandar Sundarrajan, Shashanka Puranika Kota, Naresh Kumar Mani

**Affiliations:** 1 Microfluidics, Sensors and Diagnostics (μSenD) Laboratory, Centre for Microfluidics, Biomarkers, Photoceutics and Sensors (μBioPS), Department of Biotechnology, Manipal Institute of Technology, Manipal Academy of Higher Education, Manipal, Karnataka 576104 India; 2 Department of Bioinformatics, Manipal School of Life Sciences, Manipal Academy of Higher Education, Manipal, Karnataka 576104 India

**Keywords:** Karaya gum thickener, Ratio-metric displacement-based colorimetric assay, Thread-based microfluidics, Chlor-zinc iodide, Food safety, Biochemistry, Biological techniques, Chemistry, Materials science

## Abstract

Karaya gum, a natural food-grade thickener, is an adulterant that compromises product quality and consumer safety. Herein, we develop a low-cost, rapid and portable device that employs thread-based microfluidics integrated with ratio-metric displacement to detect and quantify Karaya gum, with a limit of detection of 0.1%w/v. The interaction between the cellulose in the bamboo viscose thread and the chlor-zinc-iodide reagent causes the thread to turn blue immediately. On addition of simulated Karaya gum samples, a brown region appears beyond the blue region due to hydrogen bond formation between the hydroxyl groups of the thread and the complex sugars in the gum, thereby causing the displacement of iodine, which was confirmed by FTIR analysis. Higher gum concentrations increase viscosity and result in less displacement of iodine, with the blue-to-brown colorimetric ratio being directly proportional to the gum concentration. These observations were confirmed by measuring the viscosity of the Karaya gum and correlating it with the structure and nature of the gum. Furthermore, the variation in the iodine release was quantified using grayscale values utilizing Fiji software. The device was implemented for field trial using commercial basil drink. This approach offers an efficient alternative to conventional cumbersome detection strategies and has enormous potential for onsite food safety and quality control.

**Supplementary Information:**

The online version contains supplementary material available at 10.1038/s41598-025-19210-4.

## Introduction

Food additives (organic or synthetic) are essential for enhancing the quality, flavour, nutritional value and visual appeal of foods, along with their color, durability, and sensory acceptability^1,2^. Preservatives, sweeteners, thickeners, flavour enhancers, emulsifiers, and colourants are commonly used as additives^2,3^. Among these categories, thickeners are of special interest because of their ability to modify the rheology of food products by increasing viscosity. This characteristic feature can be attributed to the presence of polysaccharides in thickeners that swell when hydrated, as they modify the structural properties of the product^4^. Some natural sources for thickeners are animals (e.g., gelatin, chitosan), algae (e.g., agar, carrageenan), microbes (e.g., xanthan gum, salecan), plants (e.g., corn starch, rice flour) and plant exudates (e.g., gum arabic, tragacanth gum)^4,5^. Although these products can greatly improve the shelf-life of foods, they are safe only when they are used minimally.

Generally, adulteration is performed either by substituting or diluting high-quality ingredients with lower-value ingredients, which can further degrade the quality of food^6^. This often leads to reduced consumer safety as well as confidence and health issues that can range from respiratory diseases to heart attacks and even cancer in certain situations^7^. Henceforth, assessing the nature and type of food additives and their control measures is extremely important for maintaining public health and the quality of food. Karaya gum, or the exudate of plants of the genus *Sterculia* (mainly *Sterculia urens*), is mainly used in the medical field for dental usage and as a stoma sealant since Karaya gum is not digested in the human gut^5,8^. Additionally, it is used as a laxative in the pharmaceutical industry. For food applications, it is added to improve the texture, stability and quality of various food products^8,9^ including ice cream, frozen desserts, cheese spreads, and sausages^10^. Karaya gum also enhances the stability of baked products such as glazes, whipped creams, and meringues^5,11^. It also controls ice crystal formation in ice creams and sherbets, which helps improve product stability.

Karaya gum contains D-galacturonic acid, D-galactose, and L-rhamnose along with glucuronic acid as a side chain. The three sugars, as well as the partial acetylation of the polysaccharides, provide the gum’s property of gel formation instead of full solubility in water, a property that improves with increasing temperature^12–14^. According to the European Food Safety Authority, the advised daily intake of Karaya gum should not exceed 7 g/L (0.7% w/v)^15^. Some of the observed side effects include hypersensitivity, abdominal discomfort, dermatitis, gastrointestinal distress, hay fever, urticaria, asthma, and other respiratory allergies, such as coughing and wheezing^16–22^. Conventionally, Karaya gum is detected via gas chromatography (to analyse its monosaccharide composition), gel permeation chromatography, and infrared spectrometry. However, these methods have several drawbacks, such as expertise requirements, high cost, and difficulty in processing heterogeneous gums and actual food samples^15^.

Over the last two decades, microfluidic devices, often termed ‘lab-on-a-chip,’ have garnered significant attention as analytical tools because of their advantages, such as less sample consumption, reduced time involved, and better sensitivity^23–25^. The liquid usually flows laminarly in channels of a size range of micrometres. Microfluidic devices can be made of inorganic materials (such as glass, silica, ceramics, etc.), polydimethylsiloxane, rigid polymers or organic materials (such as paper^26^ and threads, which are cellulose-based)^27–36^. Thread-based microfluidic devices are widely available, inexpensive, and generally hydrophilic. In some cases where hydrophilicity must be induced, mercerisation is performed to improve the rate of reagent absorption and penetration^37^. Another advantage of thread-based microfluidic devices is that no external pumps are required as the liquid moves by capillary action or ‘wicking’. Threads have been explored for various low-cost detection and biomedical applications^38^ owing to their flexibility, mechanical strength, and simplicity of fabrication^38–44^.

**Fig. 1:**
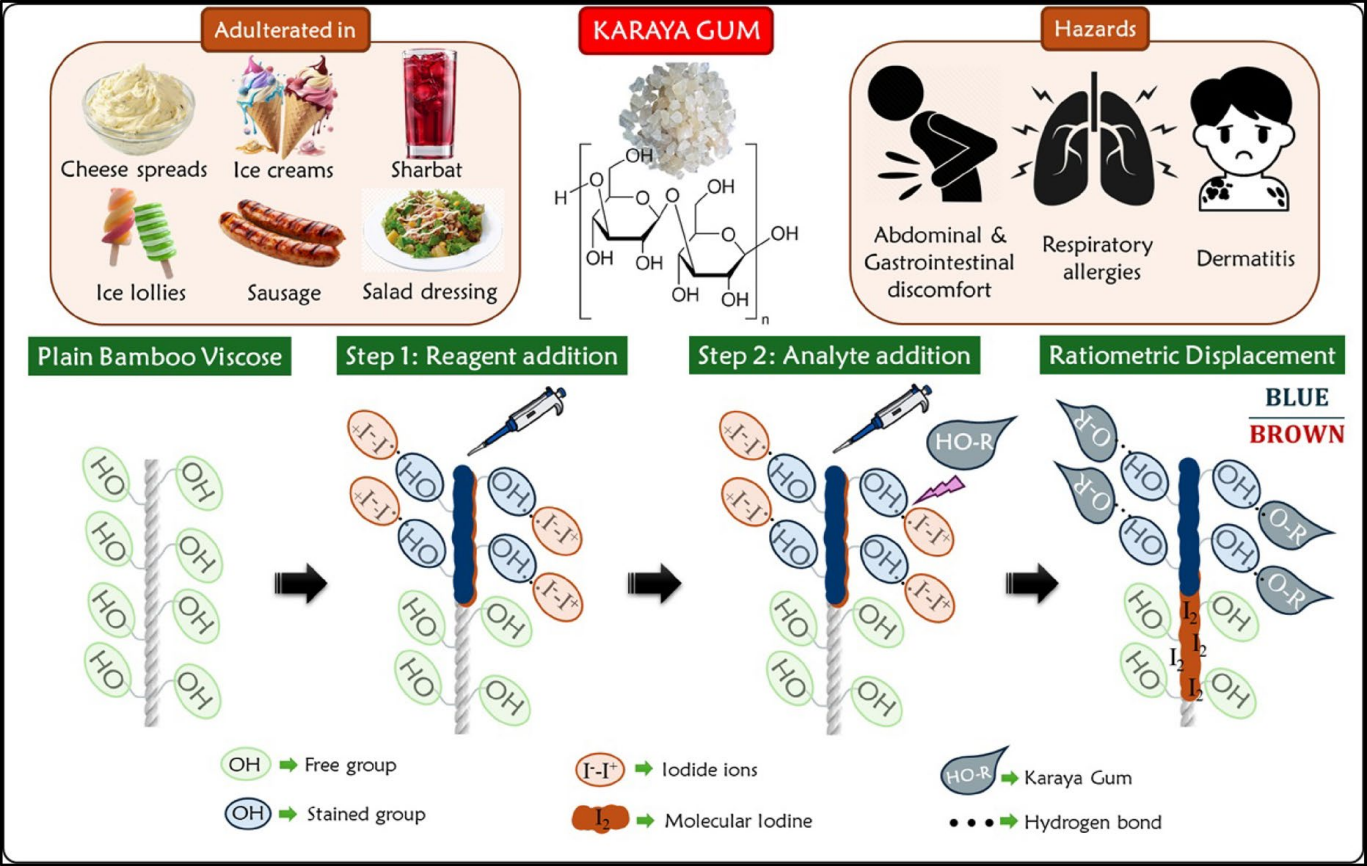
Schematic diagram of ratio-metric displacement-based detection of Karaya gum using bamboo viscose thread device.

In this work, we leveraged bamboo viscose thread combined with a novel distance-based ratio-metric assay to detect Karaya gum. We developed a robust and frugal approach by exploiting the interactions among the hydrophilic groups of the thread, the chlor-zinc-iodide reagent and the Karaya gum. First, the cellulose groups in the thread interact with the chlor-zinc-iodide reagent, resulting in a blue color. Upon the addition of Karaya gum, a secondary brown zone or region appears beyond the blue region owing to the displacement of iodine. This is due to the possible hydrogen bonding between the sugars of Karaya gum and the thread’s hydroxyl groups. Additionally, the ratio of blue to brown length can also be correlated with the viscosity of the gum (Fig. 1). To the best of our knowledge, this is the first study involving thread devices for detecting thickeners. Our method is simple, portable and inexpensive, making it an ideal substitute for conventional detection methods. This new on-spot sensing method can be an enormous step forward in detecting hazardous food additives with application in food safety and quality control.

## Results

### Standard and simulated analyses of Karaya gum utilizing Thread-based iodine displacement assay

**Fig. 2:**
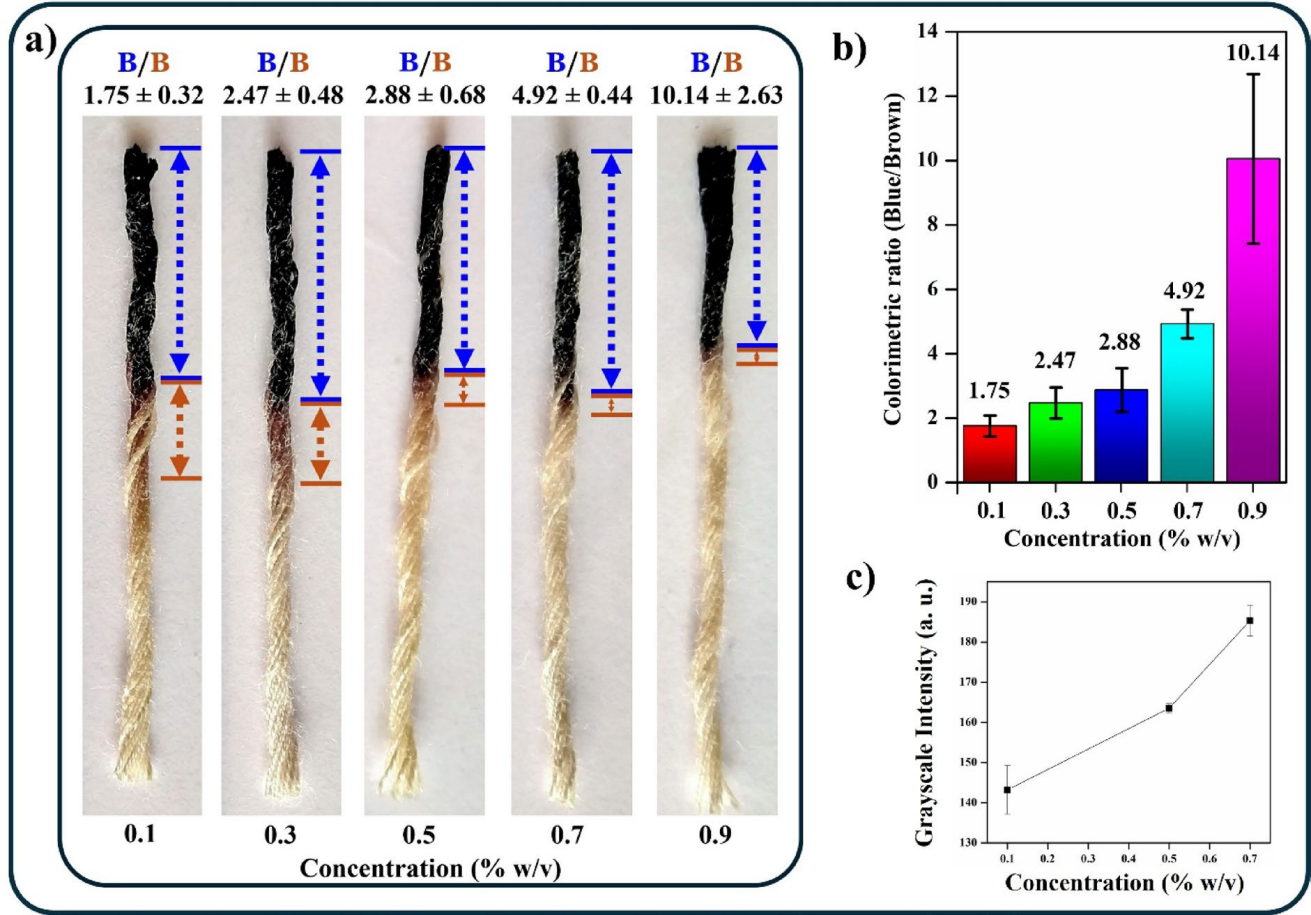
(**a**) Migration distance of iodine (brown region) after the addition of various concentrations of standard Karaya gum (blue region) in ascending order (0.1%, 0.3%, 0.5%, 0.7% and 0.9%). (**b**) Colorimetric ratio observed after the addition of different concentrations of standard Karaya gum (0.1%, 0.3%, 0.5%, 0.7% and 0.9%). (**c**) Grayscale intensity.

To detect Karaya gum, a plant-based food thickener, “bamboo viscose thread,” has been used. The material composition of the thread is Viscose 100, which denotes the presence of 100% cellulose derived from bamboo fibres that are highly hydrophilic without any binders. As a result, there is no requirement for mercerisation, thus making the fabrication of the thread-based device incredibly simple. First, a chlor-zinc-iodide (CZI) reagent was deposited or drop-cast on the bamboo viscose thread. Consequently, a blue color was observed. This was due to the formation of weak intermolecular interactions between the free OH groups in the cellulose of the bamboo viscose thread and the iodine in the CZI reagent (Fig. 2). Upon introduction of Karaya gum onto the device, a brown region (beyond the blue region) was observed on the thread. This could be due to the expulsion or displacement of iodine molecules (from the blue region) by Karaya gum. We hypothesize that the interaction between cellulose OH groups and the iodine in the CZI reagent is perturbed by the occurrence of hydrogen bonds between OH groups of the cellulose and sugar molecules in the Karaya gum. Interestingly, as the experiment was performed with different concentrations of Karaya gum, the lengths of the blue and brown regions drastically varied, as shown in Fig. 2a.

**Fig. 3:**
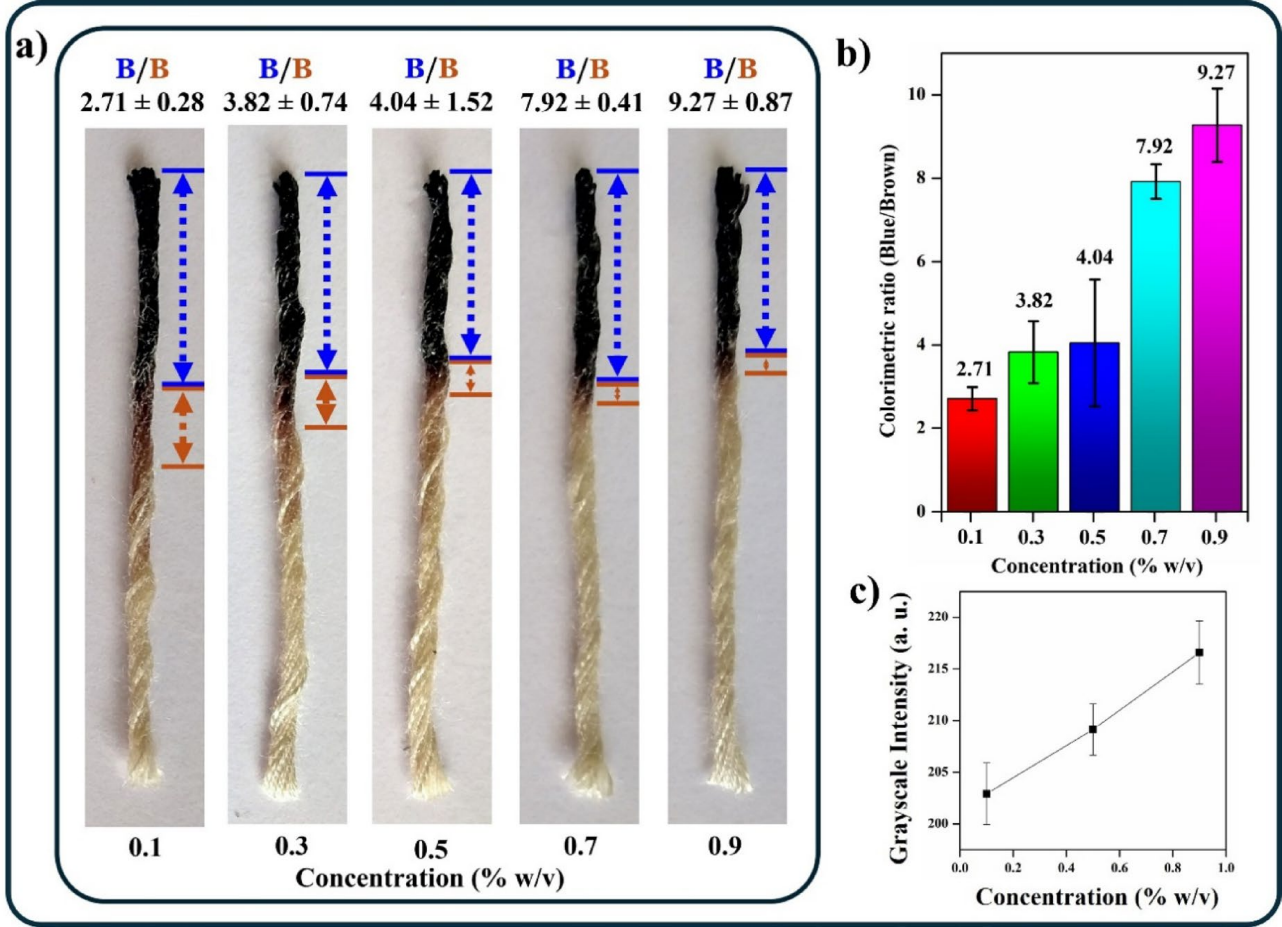
(**a**) Migration distance of the iodine (Brown region) on addition of the various concentrations of Karaya gum in simulated juice samples (Blue region) in ascending order (0.1%, 0.3%, 0.5%, 0.7% and 0.9%). (**b**) Colorimetric ratio observed on addition of the of Karaya gum in simulated juice samples (0.1%, 0.3%, 0.5%, 0.7% and 0.9%). (**c**) Grayscale intensity.

To evaluate the correlation between the concentration of Karaya gum and the iodine displaced, the lengths of the blue and brown regions in the thread images were measured using Fiji software. The results were standardised by calculating the colorimetric distance ratio of blue/brown. As the concentration of Karaya gum increased, the ratio increased, as depicted in the graph (Fig. 2b). The increase in the ratio may be attributed to the effect of the viscosity of the Karaya gum. Due to the close concentrations of Karaya gum analysed, there is a certain overlap of the ratio of distances obtained for the Karaya gum concentrations up to 0.5%w/v. However, since the acceptable limit of intake for Karaya gum is defined to be 0.7%w/v, we may clearly differentiate concentrations below and above the acceptable limit based on our developed assay. Furthermore, we measured the grayscale intensity of the brown region in Fiji software to partially quantify the iodine displaced. It can be inferred from the graph (Fig. 2c) that an increase in the Karaya gum concentration increases the grayscale intensity, thus implying lower displacement of iodine. This is also attributed to the crucial role played by the viscosity of the Karaya gum. Additionally, we simulated Gond katira juice and found that the results followed a trend similar to that of standard samples (Fig. 3a–c).

### Displacement of iodine based on the viscosity of Karaya gum

**Fig. 4:**
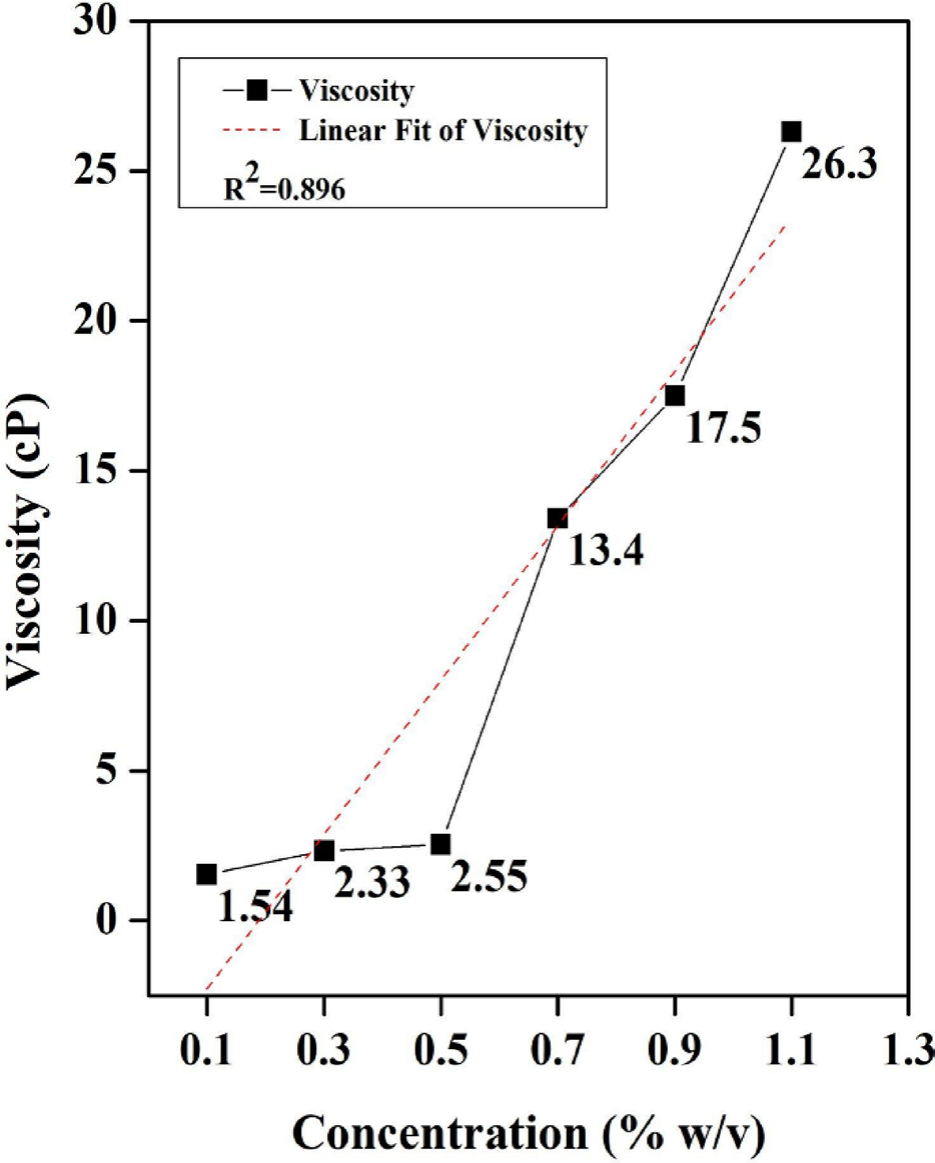
Viscosity of the Karaya gum solutions of varying concentrations (0.1%, 0.3%, 0.5%, 0.7%, 0.9% and 1.1%).

Karaya gum being hydrophilic in nature, interacts strongly with cellulose and is known to reduce the surface tension. However, due to the presence of complex macromolecules in its side chain and higher molecular weight (~ 10 million daltons), the gum molecules are adsorbed through H-bonding and van der Waals forces to the thread fibre surfaces^45,46^. Hence, this property along with the increasing viscosity of Karaya gum dominates the surface tension forces (gums generally reduce the surface tension^47^, leading to reduced displacement of iodine with increasing concentration of Karaya gum. To confirm this, the viscosities of the Karaya gum solutions were measured to assess their major influence on the assay. It is found that the viscosity is directly proportional to the concentration of Karaya gum. The data from the tests performed on the thread correlated well with the observed pattern of an increasing trend as shown in Fig. 4.

### FTIR spectral analysis

**Fig. 5:**
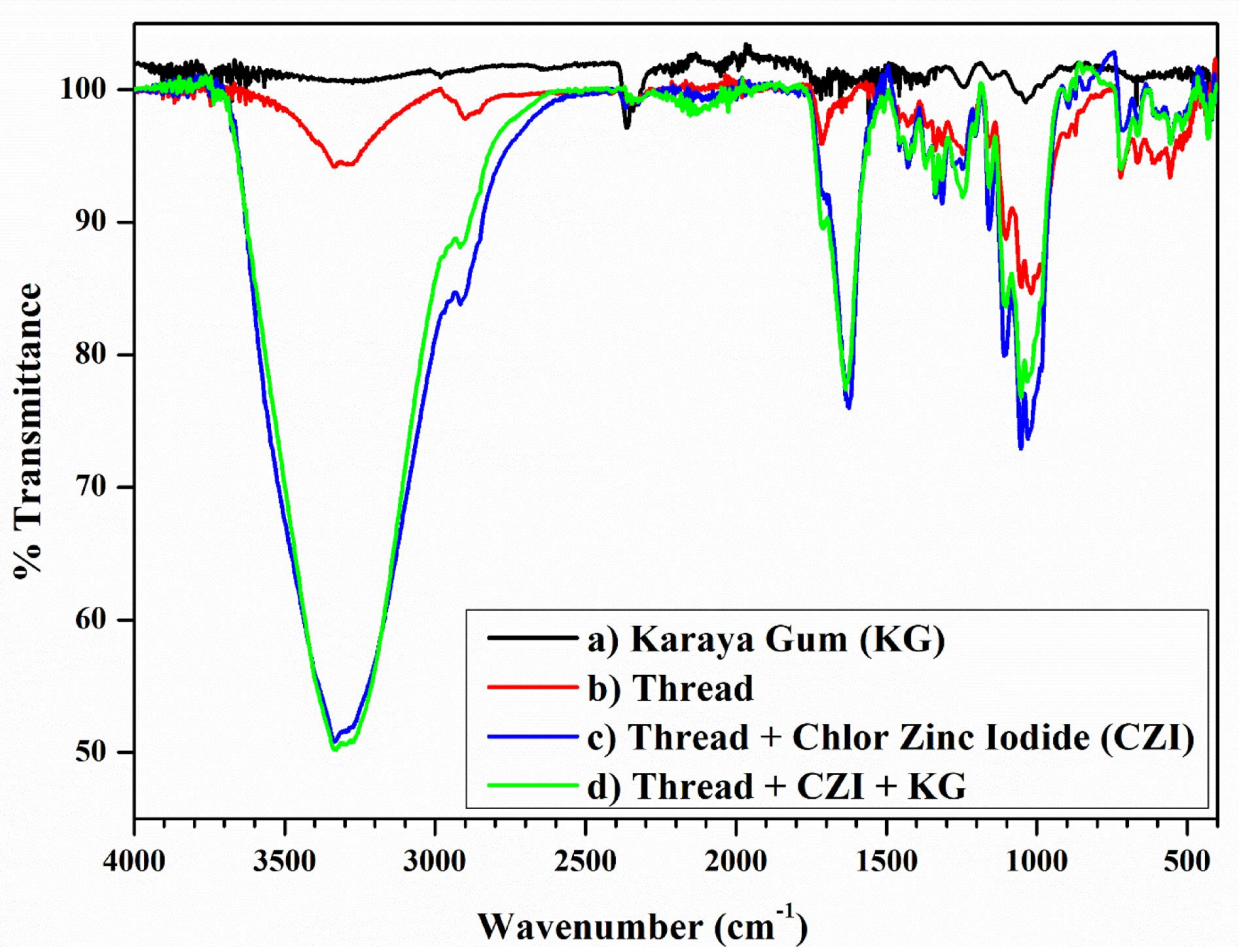
FT-IR analysis of the interaction between Karaya gum and bamboo viscose thread.

Furthermore, FT-IR analysis was conducted to assess the interaction between Karaya gum (KG) and bamboo viscose thread in the presence of a chlor-zinc-iodide (CZI) reagent (Fig. 5). A minor peak at approximately 3200–3370 cm^− 1^ indicates the availability of free OH groups from both the OH and COOH moieties of KG (a). Similarly, bamboo viscose thread (b) exhibited a band at 3300 cm^− 1^, implying the presence of free hydroxyl groups. Upon addition of the CZI reagent, the bamboo viscose thread manifests a broader band at 3345 cm^− 1^, revealing a possible intermolecular hydrogen bonding interaction between free hydroxyl groups and iodide ions through O-H…I^−^ bonding. However, KG displaced the above interaction by constructing intermolecular hydrogen bonds with bamboo viscose through O–H_cellulose_…O_KG_ bonding by expelling molecular iodine, which was revealed by a broad band at 3353 cm^− 1^. Other characteristic assignments are given in Table 1.

**Table 1:** FT-IR assignments of Karaya gum, thread and the reagents used.

Name of the chemicals	Absorption bands (cm^− 1^)	Assignments (functional groups)	Standard reference range (cm^− 1^)
Karaya gum	2945	C–H stretching	2950−2800 (alkane)
	1625	C=O stretching	1600−1400
	1150	C–O–C/C–O stretching	1200−1000
Thread	2910	C–H stretching	2950−2800 (alkane)
	1699	H–O–H bending	1700−1600
	1350	C–C stretching	1375−1320
	1100	C–O–C stretching	1090–1170
	700	Out of plan vibrations	800−600
Thread + CZI and Thread + CZI + KG	2910−2825	C–H stretching	2950−2800 (alkane)
	1635	Conjugated carbonyl stretching	1770−1620
	1200–1400	Skeletal vibration of cellulose	1200–1500
	1050	Cellulose fingerprint	1000–1100
	710	Cl–Zn–I stretching and bending vibrations	1000−500^48^

## Field trial for detecting Karaya gum in juice samples

**Fig. 6:**
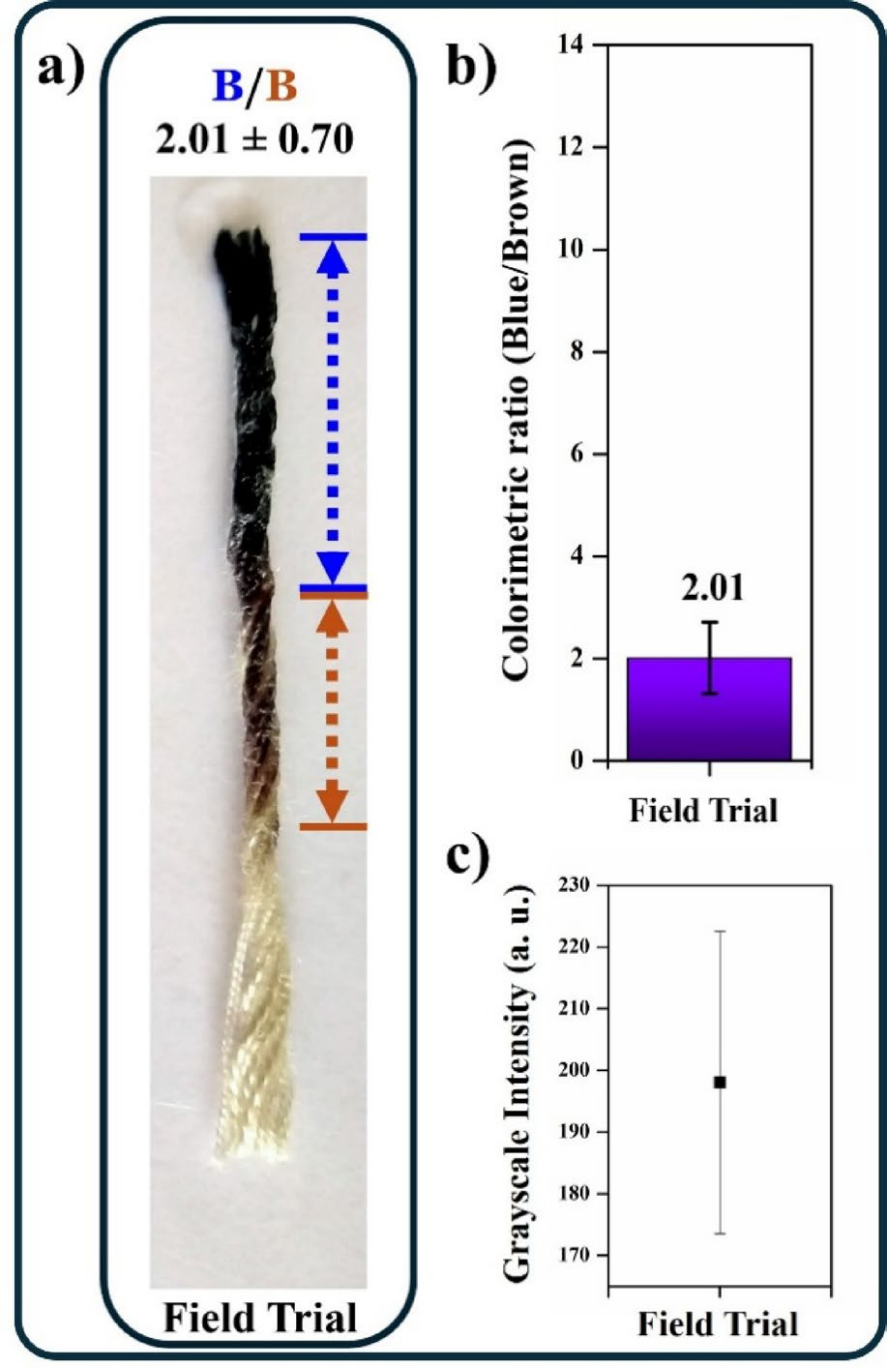
Field trial for detecting Karaya gum in juice sample.

To assess the practical utility of this approach, we conducted a field trial with a commercial basil drink (Fig. 6). Karaya gum is commonly used in sherbets and gond katira flavoured juices. The ratio of blue to brown distance for the drink was 2.01 ± 0.70 mm. The thickener concentration was deduced to be approximately between 0.1 and 0.3% w/v from the analysis results of standard Karaya gum concentrations.

## Conclusion

The results of our study show that plain bamboo viscose thread can be used as a sensitive detection and quantification tool for Karaya gum. By exploiting the thread’s natural wicking ability, and its interaction with Karaya gum polysaccharides and CZI regent, a simple distance-based ratio-metric assay was developed with limit of detection (LOD) of 1 g/L (0.1% w/v). An increase in the blue/brown ratio can be quantified visually and thus corroborates to the increasing concentration of Karaya gum. Further FTIR analysis and viscosity measurements strengthened the versatility and utility of this method, widening the scope of the distance-based ratio metric assays in terms of food safety, quality control, and compliance. This approach may eventually pave the way for easier and more effective detection of hazardous food additives. Future research in this direction may expand its application towards sensitivity enhancement and extend it to more heterogeneous food samples.

## Materials and methods

### Materials

Karaya gum was purchased from a local wholesale retailer and bamboo viscose thread (100% bamboo viscose crochet yarn, 4 ply, off-white colour, 2.5 mm thickness) from Amazon India online store (https://www.amazon.in/Crochet-Now-Bamboo-Viscose-Grams/dp/B09993MQ26). Zinc chloride and 1 N hydrochloric acid were purchased from SRL Pvt. Ltd., India. Potassium iodide was purchased from Merck, India. Iodine resublimed was purchased from Loba Chemie Pvt Ltd, India.

### Methods

#### Sample and reagent preparation

Karaya gum pieces were crushed into a fine powder and dissolved in 1 N HCl to prepare a stock solution of 1.1%w/v. Different concentrations of the gum solution (0.1, 0.3, 0.5, 0.7 and 0.9%w/v) were further prepared from this stock solution. 10 g of Zinc chloride (ZnCl_2_) and 3.25 g of Potassium iodide (KI) were taken in an amber coloured falcon and dissolved in 2 mL of water, followed by mixing using a vortex. Later, 0.25 g of iodine resublimed was added to the solution to achieve a final concentration of 1.9 g/mL ZnCl_2_, 0.62 g/mL KI and 0.05 g/mL iodine resublimed in 5.25 mL of water. It was mixed vigorously for 15 min for obtaining the final Chlor-zinc-iodide reagent. The reagent was then stored in the refrigerator at 4˚C until further use and in the ice bucket while performing the experiments.

#### Standard analysis of Karaya gum

The thread-based microfluidic device was fabricated by cutting the bamboo viscose thread into 5 cm pieces and placed on pieces of transparent polyester film sheets. 20 µL of the CZI reagent was dropped at one end of the thread, allowing the absorption of the reagent. The transparency film sheet with the instantly blue stained threads were stored in glass petri plates and refrigerated at 4 °C for 10 min. Next, the test reagent embedded threads were removed out, and 30 µL samples of different concentrations of Karaya gum were added immediately at the same end of the thread. The threads were observed for the appearance of brown region exactly after 1 min and images were captured using a smartphone (Oppo A5 2020, 12 MP, HDR mode) placed at a fixed distance (12 cm) from the thread, utilizing natural light. Three trials of the experiments were carried out for each concentration of the gum to check the reproducibility of the assay. The lengths of the blue and brown regions on the thread were measured with Fiji software to calculate the colorimetric distance ratio. Additionally, the intensity of the brown regions was partially quantified by obtaining grayscale values to confirm the iodine displacement. The limit of detection (LOD) was determined based on the visual observation of the minimum distinguishable concentration through the blue and brown regions.

#### Viscosity measurement of the Karaya gum samples

The viscosity of the Karaya gum samples was measured at 100 rpm using a digital rotational viscometer (LMDV200, Bio Bee Tech, India) in order to understand the influence of viscosity on the iodine displacement in the thread.

#### FT-IR analysis of the molecular interactions between the thread-based device and analyte

The functional groups involved in the interaction between Karaya gum, bamboo viscose thread and the CZI reagent were assessed via an FTIR spectrometer (IRSPIRIT QATR-S, Shimadzu, India) in the ATR mode (in the range of 400–4000 cm^− 1^).

#### Simulated analysis of Karaya gum

Gond katira juice was simulated following a commonly available recipe with basic components of beverages containing Karaya gum or gelling agent, sugar, salt, chia seeds and purified water. One teaspoon each of Karaya gum powder and chia seeds was taken and soaked separately in 30 mL of purified water overnight. One teaspoon of soaked Karaya gum was added to an empty beaker, followed by one teaspoon of soaked chia seeds, a pinch of salt and sugar, and 30 mL of water. The ingredients were mixed properly by stirring, and the sample was analysed using our developed distance-based ratio-metric thread device. All the trials were done in triplicates and standard deviation (SD) was measured.

#### Field trial

Commercial basil seed drink was procured from a local general store in Manipal, Udupi district, South Karnataka region of India. 30 µL of the drink was added to the end of our developed blue colour-stained thread device, and the distance of iodine displacement was measured in Fiji software from the captured images.

## Supplementary Information

Below is the link to the electronic supplementary material.

Supplementary Material 1

## Data Availability

The datasets used and/or analyzed during the current study are available from the corresponding author on reasonable request.
