# Supplementary material for: Distance-based ratio metric assay using thread-based devices for Karaya gum detection in food samples
Source: Sci Rep. 2025 Sep 29;15:33626. doi: 10.1038/s41598-025-19210-4 (PMC12480264; doi:10.1038/s41598-025-19210-4)
Supplement: Supplementary file 1 — Supplementary Material 1 [file 41598_2025_19210_MOESM1_ESM.docx]

SUPPLEMENTARY

| **Method** | **Detection Time** | **Sample Pretreatment** | **Cost per Detection** | **Expertise required** | **Portability** | **Reference** |
| --- | --- | --- | --- | --- | --- | --- |
| Traditional Methods (GC, GPC) | Generally time-consuming due to complex procedures. | Difficulty in processing heterogeneous gums and actual food samples, | Significantly more expensive.  GC: $8.57  GPC: $79.97 | Requires professional expertise to operate the equipment and interpret data. | Lab-based, cumbersome instrumentation. | (ANS), E. P. on F. A. and N. S. added to F. et al. Re-evaluation of karaya gum (E 416) as a food additive. EFSA J. 14, e04598 (2016).  Mass spectrometry/Chromatography Techniques. *National Chemical Laboratory* Available at: https://www.ncl-india.org/files/Analytical/MSChromatographyTechniques.aspx. (Accessed: 13th August 2025) |
| Proposed Thread-based Assay | 1 minute | No pretreatment.  For the field trial, the commercial drink was used directly. | Inexpensive: $0.004 ≈ $0 | Simple procedure, suitable for on-site/field use. | Highly portable, uses smartphone for imaging. | Our work |

Current conversion (as of 13/08/2025) where ₹1 = $0.011
